# Proteome Analysis of Urinary Biomarkers in a Bovine IRBP-Induced Uveitis Rat Model *via* Data-Independent Acquisition and Parallel Reaction Monitoring Proteomics

**DOI:** 10.3389/fmolb.2022.831632

**Published:** 2022-02-22

**Authors:** Weiwei Qin, Xuyan Qin, Lujun Li, Youhe Gao

**Affiliations:** ^1^ Department of Anesthesiology, Qingdao Municipal Hospital, Qingdao University, Qingdao, China; ^2^ Department of Biochemistry and Molecular Biology, Gene Engineering Drug and Biotechnology Beijing Key Laboratory, Beijing Normal University, Beijing, China; ^3^ Department of Dermatology, Qingdao Hiser Hospital Affiliated to Qingdao University, Qingdao, China

**Keywords:** urine proteome, experimental autoimmune uveitis (EAU), biomarker, data-independent acquisition (DIA), parallel reaction monitoring (PRM)

## Abstract

Uveitis, a group of intraocular inflammatory diseases, is one of the major causes of severe visual impairment among the working-age population. This study aimed to screen potential urinary biomarkers for uveitis based on proteome analysis. An experimental autoimmune uveitis (EAU) rat model induced by bovine interphotoreceptor retinoid-binding protein (IRBP) was used to mimic uveitis. In discovery phase, a total of 704 urinary proteins were identified via data-independent acquisition (DIA) proteomic technique, of which 76 were significantly changed (34, 36, and 37 on days 5, 8, and 12, respectively, after bovine IRBP immunization). Gene Ontology annotation of the differential proteins indicates that acute-phase response, innate immune response, neutrophil aggregation, and chronic inflammatory response were significantly enriched. Protein-protein interaction network indicates that these differential urinary proteins were biologically connected in EAU, as a group. In validation phase, 17 proteins having human orthologs were verified as the potential markers associated with uveitis by parallel reaction monitoring (PRM) targeted quantitative analysis. Twelve differential proteins changed even when there were no clinical manifestations or histopathological ocular damage. These 12 proteins are potential biomarkers for early diagnosis of uveitis to prevent the development of visual impairment. Five differential proteins changed at three time-points and showed progressive changes as the uveitis progressed, and another five differential proteins changed only on day 12 when EAU severity peaked. These 10 proteins may serve as potential biomarkers for prognostic evaluation of uveitis. Our findings revealed that the urinary proteome could sensitively reflect dynamic pathophysiological changes in EAU, and represent the first step towards the application of urinary protein biomarkers for uveitis.

## Introduction

Uveitis is a group of intraocular inflammatory diseases that mainly involve the uvea, the blood vessel-rich pigmented middle layer of the wall of the eye. Uveitis carries a high risk of vision loss and usually affects people aged 20–50 years (Rothova et al., 1992; Gritz and Wong, 2004; Krishna et al., 2017). In developing countries, approximately 5–25% of irreversible blindness is caused by uveitis and its complications (Rao, 2013; Tsirouki et al., 2018). In addition to infectious agents, autoimmune reactions are one of the main causes of uveitis. For autoimmune uveitis, corticosteroids and immunomodulatory agents are the mainstay of treatment (Larson et al., 2011). However, there are some uveitis patients who fail to respond to treatment. And it is common for the recurrence to occur during the tapering phase of corticosteroid uses. In addition, long-term treatments may have several intraocular and systemic side effects, such as high intraocular pressure, sterile endophthalmitis, and nephrotoxicity (Jonas et al., 2003; Moshfeghi et al., 2003).

The diagnosis and differential diagnosis of uveitis remain challenging even to experienced specialists (Herbort et al., 2009; Sève et al., 2017) because of the profound clinical overlap between uveitis entities induced by different etiologies, the limited availability of clinical specimens and the limited reliability/accuracy of currently available tests. Timely diagnosis of non-autoimmune uveitis, in particular infectious uveitis and masquerade syndrome, can be difficult. Therefore, the identification of noninvasive and accurate biomarkers for diagnosis and/or disease monitoring is of great clinical significance. If uveitis is diagnosed earlier, effective measures may be used to prevent vision loss.

Urine is a promising resource for biomarker research and is attracting more and more attention. Without strict homeostatic regulation, urine can sensitively reflect changes in the body at an early stage (Gao, 2013; Li et al., 2014). Mass spectrometry-based proteomics have dramatically improved and emerged as a prominent tool in the field of biomarker studies. Many candidate biomarkers for uveitis have been reported primarily in the blood, tears, and aqueous humor (Curnow and Murray, 2006; Guo et al., 2015; Pepple et al., 2015; Walscheid et al., 2015). Urinary proteomic studies have identified many candidate biomarkers for autoimmune inflammatory diseases, such as rheumatoid arthritis, autoimmune myocarditis, and inflammatory bowel disease (Shao et al., 2011; Qin et al., 2019a). However, there have been limited studies of protein biomarkers for uveitis in urine compared with the wide application of urinary protein markers for other diseases.

Experimental autoimmune uveitis (EAU) is a T cell-mediated autoimmune disease that targets the neural retina and related tissues (Caspi et al., 1986; Lee et al., 2014). It is an induced noninfectious type of uveitis, as opposed to a spontaneous, autoimmune disease model. The hallmarks of EAU are the onset of ocular inflammation, disruption of the retinal architecture, and partial to complete destruction of the photoreceptor cell layer (Caspi, 2003). It shares many common features in clinical and histological aspects with human uveitis (Agarwal et al., 2012). Susceptibility to EAU varies between rat strains. In this study, Lewis rats which develops characteristically severe uveitis were used. Others works in the same experimental context using Wistar Rat (Arroul-Lammali et al., 2012). The urine proteome is affected by multiple factors, such as age, diet, exercise, gender, medication, and daily rhythms (Wu and Gao, 2015). Animal models can be used to minimize the impact of many uncertain factors by establishing a direct relationship between a disease and corresponding changes in urine (Gao, 2014).

**FIGURE 1:**
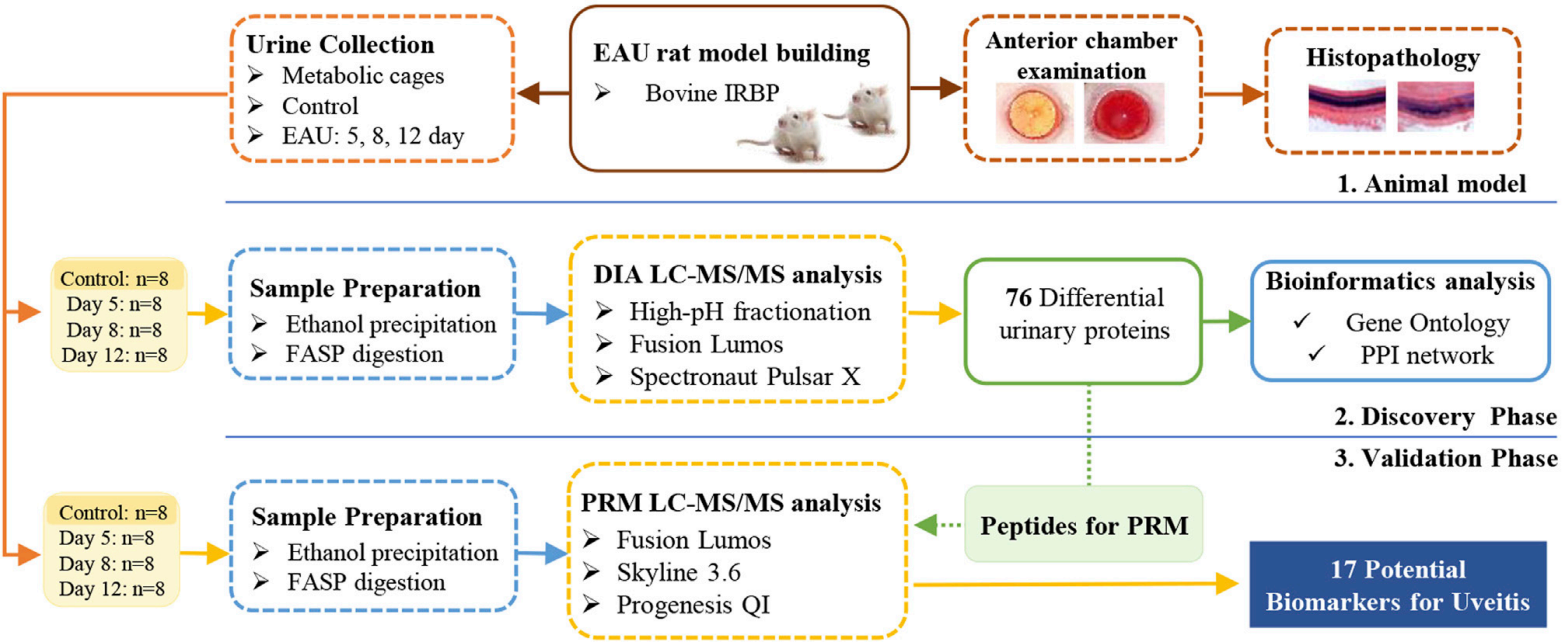
Workflow of the study of urine proteome changes in experimental autoimmune uveitis (EAU) rats.

This study aimed to identify potential urinary protein biomarkers related to uveitis by using the bovine IRBP-induced uveitis rat model. The experiment was conducted in two phases, as shown in Figure 1. In the discovery phase, a data-independent acquisition (DIA) approach was used to profile the proteome of urine from EAU rats and compared with that of controls. In the validation phase, the differentially changed proteins were validated by parallel reaction monitoring (PRM) targeted quantitative analysis using a quadrupole-orbitrap mass spectrometer.

## Materials and Methods

### Animals and Uveitis Induction

Fifty male Lewis rats (160–180 g) were purchased from Charles River China (Beijing, China). All animals were maintained on a standard laboratory diet under controlled indoor temperature (21 ± 2°C), humidity (65–70%) and 12 h light–dark cycle conditions. All experiments were performed using protocols approved by the Animal Care and Use Committee of the Qingdao Municipal Hospital Medical Ethics Committee. All methods were carried out in accordance with relevant guidelines and regulations of the National Health Commission and the Ministry of Science and Technology.

The Lewis rats were randomly divided into two groups: a control group (n = 25) and an EAU group (n = 25). EAU was induced according to the procedure described previously (Caspi, 2003). Briefly, rats in the EAU group were immunized with 30 µg of bovine IRBP peptide R16 (ADGSSWEGVGVVPDV, BGI, Beijing, China), emulsified 1:1 in complete Freund’s adjuvant (Sigma-Aldrich, St. Louis, MO, United States). The rats in the control group were given a subcutaneous injection of the same volume of CFA saline.

The experiment was conducted in two phases, for details, see Figure 1. For the discovery phase, differentially changed urinary proteins were identified by DIA quantification in 32 independent samples from the control group (eight samples) and the EAU group on days 5, 8 and 12 (eight samples per time point); for the validation phase, the 32 remaining urine samples (8 from the control group and the others collected from the EAU group on days 5, 8 and 12, with 8 samples per time point) were evaluated by PRM targeted quantification.

### Histological Analysis of EAU

For histopathology, three rats in the experimental group and three rats in the control group were randomly sacrificed at 8, 12 and 16 days after immunization injection. Their eyes were harvested and then quickly fixed in 10% neutral-buffered formalin. The formalin-fixed tissues were embedded in paraffin, sectioned (4 mm) and stained with hematoxylin and eosin (H&E) to reveal histopathological lesions.

### Urine Collection and Sample Preparation

Urine samples were collected from the control and experimental groups at days 5, 8, 12 and 16 after immunization injection. Rats were individually placed in metabolic cages for 6 h. During urine collection, no food was provided to the rats to avoid urine contamination. After collection, the urine samples were immediately centrifuged at 2,000 g for 30 min at 4°C and then stored at −80°C.

Urinary protein extraction: 0.5 ml urine was centrifuged at 12,000 g for 30 min at 4°C. Six volumes of prechilled ethanol were added after removing the pellets, and the samples were precipitated at 4°C overnight. Then, lysis buffer (8 mol/L urea, 2 mol/L thiourea, 50 mmol/L Tris, and 25 mmol/L DTT) was used to dissolve the pellets. The protein concentration of each sample was measured by the Bradford protein assay.

Tryptic digestion: The proteins were digested with trypsin (Promega, United States) using filter-aided sample preparation methods (Wiśniewski et al., 2009). Briefly, 100 µg of the protein sample was loaded onto the 10-kD filter unit (Pall, United States). The protein solution was reduced with 4.5 mM DTT for 1 h at 37°C and then alkylated with 10 mM indoleacetic acid for 30 min at room temperature in the dark. The proteins were digested with trypsin (enzyme-to-protein ratio of 1:50) for 14 h at 37°C. The peptides were desalted on Oasis HLB cartridges (Waters, United States) and lyophilized for trap column fractionation and LC-MS/MS analysis.

### Spin Column Separation

A high-pH reversed-phase peptide fractionation kit (Thermo Pierce, United States) was used according to the manufacturer’s instructions. A step gradient of increasing acetonitrile concentrations was applied to the column to elute bound peptides. Ten different fractions were collected by centrifugation, including the flow-through fraction, the wash fraction and eight step gradient sample fractions (5, 7.5, 10, 12.5, 15, 17.5, 20 and 50% acetonitrile). The fractions were analyzed by LC-MS/MS.

### LC-MS/MS Setup for DDA and DIA

An Orbitrap Fusion Lumos Tribrid mass spectrometer was coupled with an EASY-nLC 1000 HPLC system (Thermo Scientific, Germany). For DDA-MS and DIA-MS modes, the same LC settings were used. The digested peptides were loaded onto a trap column (75 μm × 2 cm, 3 μm, C18, 100 Å). The eluent was transferred to a reversed-phase analytical column (50 μm × 250 mm, 2 μm, C18, 100 Å). The eluted gradient was 5–30% buffer B (0.1% formic acid in 99.9% acetonitrile; flow rate of 0.4 μl/min) for 60 min. The parameters were set as follows: the full scan was acquired from 350 to 1,550 m/z at 60,000, the cycle time was set to 3 s (top speed mode), the auto gain control (AGC) was set to 1e6, and the maximum injection time was set to 50 ms. MS/MS scans were acquired in the Orbitrap at a resolution of 15,000 with an isolation window of 2 Da and collision energy at 32% (HCD); the AGC target was set to 5e4, and the maximum injection time was 30 ms.

For DIA-MS acquisition, the variable isolation window DIA method with 26 windows was developed (Supplementary Table S1). The full scan was set at a resolution of 60,000 over an m/z range of 350 to 1,200, followed by DIA scans with a resolution of 30,000, HCD collision energy of 32%, AGC target of 1e6 and maximal injection time of 50 ms. A quality control DIA analysis of the pooled sample was inserted after every 8 urine samples were tested.

### LC-MS/MS Setup for PRM

Orbitrap Fusion Lumos Tribrid mass spectrometer coupled with an EASY-nLC 1200 HPLC system was used. The peptides were loaded on a reversed-phase trap column (75 μm × 2 cm, 3 μm, C18, 100 Å, Thermo Scientific, Germany), and the eluent was then transferred to a reversed-phase analytical column (50 μm × 150 mm, 2 μm, C18, 100 Å, Thermo Scientific, Germany). The elution gradient consisted of 5–35% buffer B (0.1% formic acid in 80% acetonitrile; flow rate 0.3 μl/min) for 120 min. The MS/MS scans were acquired in the Orbitrap at a resolution of 30,000 with an isolation window of 1.6 Da and collision energy at 30% (HCD), the AGC target was set to 5e4, and the maximum injection time was 60 ms.

For the PRM-MS method, thirty-two individual samples were analyzed in PRM mode. Finally, 150 peptides were scheduled, and the retention time (RT) segment was set to 8 min for each targeted peptide (Supplementary Table S2). The normalized collision energy was fixed to 30% and the quadrupole isolation window to 1.6 Da. The other parameters were the same as described in the last paragraph.

### DIA Quantification Analysis

The raw data files acquired for the ten fractions in DDA mode were processed using Proteome Discoverer (version 2.3; Thermo Scientific, Germany) with SEQUEST HT against the SwissProt Rattus database (released in May 2019, containing 8086 sequences) appended with the iRT peptide sequences. The search parameters consisted of a parent ion mass tolerance of 10 ppm; fragment ion mass tolerance of 0.02 Da; fixed modification of carbamidomethylated cysteine (+58.00 Da); and variable modifications of oxidized methionine (+15.995 Da) and deamidated glutamine and asparagine (+0.984 Da). For other settings, the default parameters were used.

The raw DIA-MS files were imported into Spectronaut Pulsar with the default settings. In brief, mass calibration was set to local mass calibration. Cross-run normalization was enabled to correct for systematic variance in LC-MS performance, and a local normalization strategy was used (Callister et al., 2006). Protein inference, which gave rise to the protein groups, was performed on the principle of parsimony using the ID picker algorithm as implemented in Spectronaut Pulsar (Zhang et al., 2007).

### PRM Quantification Analysis

Skyline (version 19.1.0.193) (MacLean et al., 2010) was used to build the spectrum library and filter peptides for PRM analysis. For each targeted protein, 2‒6 associated peptides were selected using the following rules: i) identification in the untargeted analysis with a q value <1%, ii) completely digested by trypsin, iii) containing 8–18 amino acid residues, iv) exclusion of the first 25 amino acids at the N-terminus of proteins, and v) fixed carbamidomethylation of cysteine. Prior to individual sample analysis, pooled peptide samples were subjected to PRM experiments to refine the target list. Finally, forty-four proteins with 150 peptides (Supplementary Table S2) were scheduled.

All of the PRM-MS data were processed with Skyline. Transition settings: precursor charges +2, +3; ion charge +1; ion type b, y, p; product ions from ion three to last ion −1; auto-select all matching transitions; ion match tolerance 0.02 m/z; pick 6 most intense product ions. Prior to the statistical analysis, the quantified protein intensities were normalized according to the summed intensity.

### Bioinformatics Analysis

Bioinformatics analysis was carried out to better study the biological function of the differential proteins. GO analysis was performed on the differentially altered urinary proteins identified at the discovery phase (http://www.geneontology.org/) (Ashburner et al., 2000; Consortium, 2019). In this study, significant GO enrichment was defined at *p* < 0.05.

### Data Availability.

All raw files were uploaded at www.iprox.org. URL: https://www.iprox.org/page/SSV024. html;url=15776233949742GmC; Passwords: yKSu.

## Results and Discussion

### Characterization of EAU Rats

**FIGURE 2:**
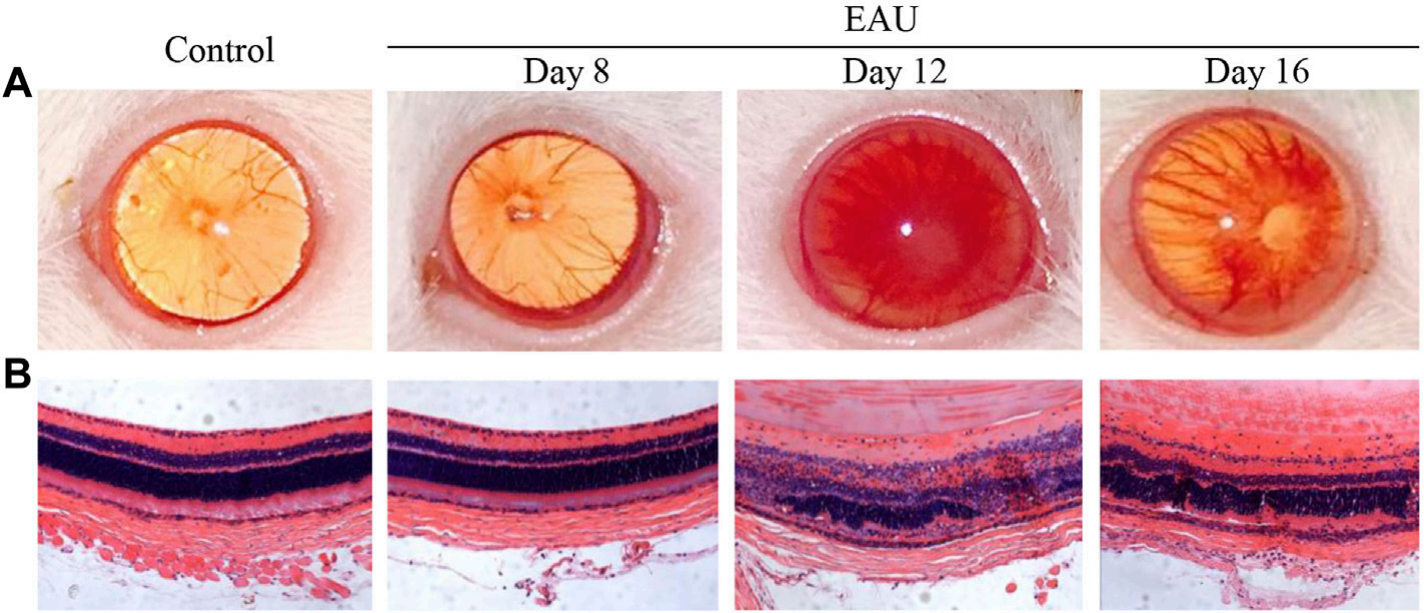
Clinical manifestations and histopathological ocular damage in EAU eyes. **(A)**: Clinical appearance upon anterior chamber examination. **(B)**: Hematoxylin and eosin staining (H&E) of retinal tissue (×20). Control: Normal control rat eye; Day 8: 8 days after bovine IRBP immunization; Day 12: 12 days after bovine IRBP immunization; Day 16: 16 days after bovine IRBP immunization.

An anterior chamber examination and histopathology analysis were performed to evaluate the progression of EAU in the Lewis rats (Figure 2). On day 8, there was no difference between the EAU and the control rats. Anterior chamber examination showed a translucent appearance, the pupil and iris blood vessels were clearly visible, and the vessels were not congested (Figure 2A). HE staining showed that the retina presented ordered retinal layers (Figure 2B). On day 12, there were obvious signs of uveitis compared with control rats. The eyeball appeared larger due to swelling and proptosis, the red reflex was absent and the pupil was obscured (Figure 2A). HE staining showed that the retinal architecture was disorganized, massive inflammatory cells infiltrated throughout the retina and choroid, and photoreceptor cells were damaged (Figure 2B). On day 16, mild vitritis and retinal folds were observed (Figure 2). These clinical manifestations and pathological changes revealed the successful generation of bovine IRBP-induced uveitis model. EAU severity peaked on day 12 after the immunization injection.

### Dynamic Urine Proteome Changes in EAU Rats

To preliminarily investigate how the urine proteome changes with EAU progression, samples from the control group and from three time points in the EAU group (days 5, 8, and 12) were analyzed via the DIA workflow. A total of 704 proteins were identified with FDR < 0.01. Among these, 76 proteins significantly changed in EAU rats compared to control rats (1.5-fold change, adjust *p* < 0.05; Table 1). At day 5, 34 differential urinary proteins, 18 of which increased and 16 of which decreased, were identified. At day 8, 36 differential urinary proteins, 17 of which increased and 19 of which decreased, were identified. At day 12, 31 differential urinary proteins, six of which increased and nine of which decreased, were identified.

**TABLE 1:** Dynamic urine proteome changes in EAU rats identified by DIA LC-MS/MS.

UniProt ID	Protein name	Fold change		
		D5	D8	D12
Q62714	Neutrophil antibiotic peptide NP-4	4.34	3.01	2.59
P02764	Alpha-1-acid glycoprotein	3.94	1.98	1.68
P30152	Neutrophil gelatinase-associated lipocalin	2.91	2.24	2.45
P06866	Haptoglobin	2.73	1.80	1.65
O55006	Protein RoBo-1	2.53	1.97	1.79
P08932	T-kininogen 2	2.48	1.71	1.63
P01048	T-kininogen 1	2.18	1.73	1.77
O88766	Neutrophil collagenase	2.15	2.39	2.36
P07647	Submandibular glandular kallikrein-9	0.60	0.65	0.63
Q62930	Complement component C9	2.02	1.56	
Q63515	C4b-binding protein beta chain	1.79	1.57	
Q6IE51	Serine protease inhibitor Kazal-type 7	0.64	0.61	
O54858	Carboxypeptidase Z	0.59	0.52	
O54728	Phospholipase B1, membrane-associated	0.45	0.44	
P62836	Ras-related protein Rap-1A	0.40	0.50	
P16573	Carcinoembryonic antigen-related cell adhesion molecule 1	1.72		1.59
Q3KRD8	Eukaryotic translation initiation factor 6		2.08	3.78
Q4KLZ6	Triokinase/FMN cyclase		1.56	1.57
Q63060	Glycerol kinase		1.67	1.67
P50115	Protein S100-A8		1.60	1.94
P82995	Heat shock protein HSP 90-alpha		0.10	0.08
P21744	Insulin-like growth factor-binding protein 4		0.61	0.63
P09006	Serine protease inhibitor A3N	1.88		
D3ZTV3	Leucine-rich repeat transmembrane protein FLRT2	1.82		
P08721	Osteopontin	1.68		
P41516	DNA topoisomerase 2-alpha	1.62		
P08649	Complement C4	1.61		
P20059	Hemopexin	1.59		
Q8K4D8	Aldehyde dehydrogenase family 1 member A3	1.51		
P19804	Nucleoside diphosphate kinase B	0.65		
Q09030	Trefoil factor 2	0.62		
P49744	Thrombospondin-4	0.56		
Q64573	Liver carboxylesterase 4	0.55		
Q63355	Unconventional myosin-Ic	0.54		
P40241	CD9 antigen	0.48		
P34901	Syndecan-4	0.34		
Q99MZ8	LIM and SH3 domain protein 1	0.34		
Q03191	Trefoil factor 3	0.33		
P19132	Ferritin heavy chain	0.30		
Q9WVH8	Fibulin-5	0.29		
Q8CJD3	Zymogen granule membrane protein 16		2.08	
Q9JLJ3	4-trimethylaminobutyraldehyde dehydrogenase		1.89	
Q99068	Alpha-2-macroglobulin receptor-associated protein		1.60	
Q9QZ76	Myoglobin		0.63	
D3ZUC6	Protein TOPAZ1		0.64	
O89117	Beta-defensin 1		0.64	
P35859	Insulin-like growth factor-binding protein complex acid labile subunit		0.62	
Q99PW3	Sialidase-1		0.62	
P38438	TGF-beta receptor type-2		0.61	
P08753	Guanine nucleotide-binding protein G(k) subunit alpha		0.57	
P04073	Gastricsin		0.54	
Q63207	Coagulation factor X		0.52	
P17559	Uteroglobin		0.46	
O88917	Adhesion G protein-coupled receptor L1		0.40	
P08723	Prostatic spermine-binding protein		0.22	
Q6P6V0	Glucose-6-phosphate isomerase			3.98
P00731	Carboxypeptidase A1			3.59
P50282	Matrix metalloproteinase-9			2.55
Q63532	Cornifin-A			2.46
P51635	Aldo-keto reductase family 1 member A1			2.40
Q5XI95	Alcohol dehydrogenase 6			1.91
P62963	Profilin-1			1.88
Q07936	Annexin A2			1.74
Q5BJY9	Keratin, type I cytoskeletal 18			1.73
Q920R3	Acyl-CoA ()-desaturase			1.73
Q5BKD0	Inactive 2′-5′-oligoadenylate synthase 1B			1.69
P10247	H-2 class II histocompatibility antigen gamma chain			1.61
P61589	Transforming protein RhoA			1.58
P63095	Guanine nucleotide-binding protein G(s) subunit alpha isoforms short			1.56
P04785	Protein disulfide-isomerase			1.53
Q793F9	Vacuolar protein sorting-associated protein 4A			1.53
P53369	7,8-dihydro-8-oxoguanine triphosphatase			1.53
Q6AYA5	Transmembrane protein 106B			1.51
P07171	Calbindin			0.62
Q4FZU2	Keratin, type II cytoskeletal 6A			0.59
P10760	Adenosylhomocysteinase			0.56

**FIGURE 3:**
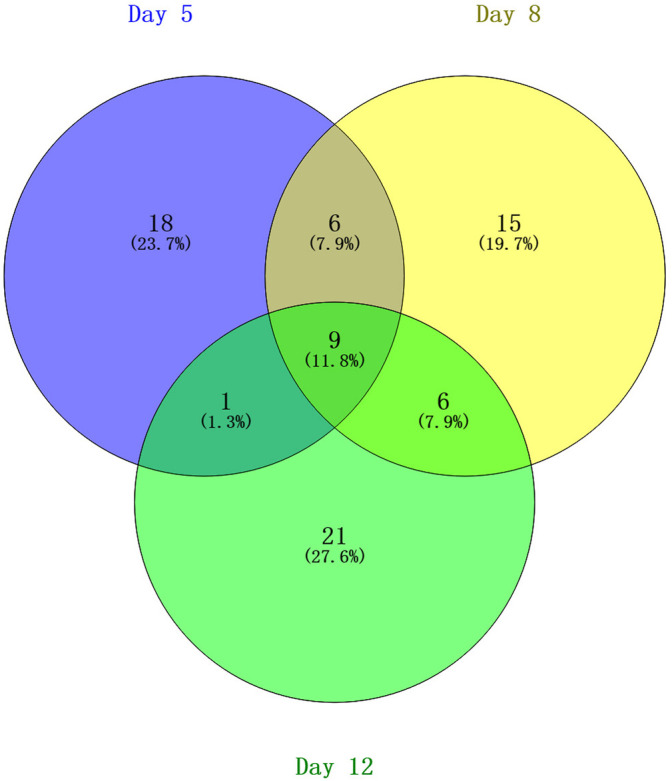
Venn diagram of the differential urinary proteins on days 5, 8, and 12 in EAU rats compared with control rats.

The overlap of these 76 differential proteins identified in EAU rats is shown with a Venn diagram (Figure 3). Fifteen urinary proteins changed significantly both on days 5 and 8, when there were no clinical manifestations and histopathological ocular damage. This suggests the potential for these urinary proteins to be used for the early detection of uveitis. Among these differential proteins, nine proteins, such as DEF4, A1AG, NGAL, HPT, ROB1, KNT2, KNT1, MMP8 and KLK9, consistently changed on day 12 when EAU severity peaked.

### Gene Ontology Analysis of Differential Proteins

**FIGURE 4:**
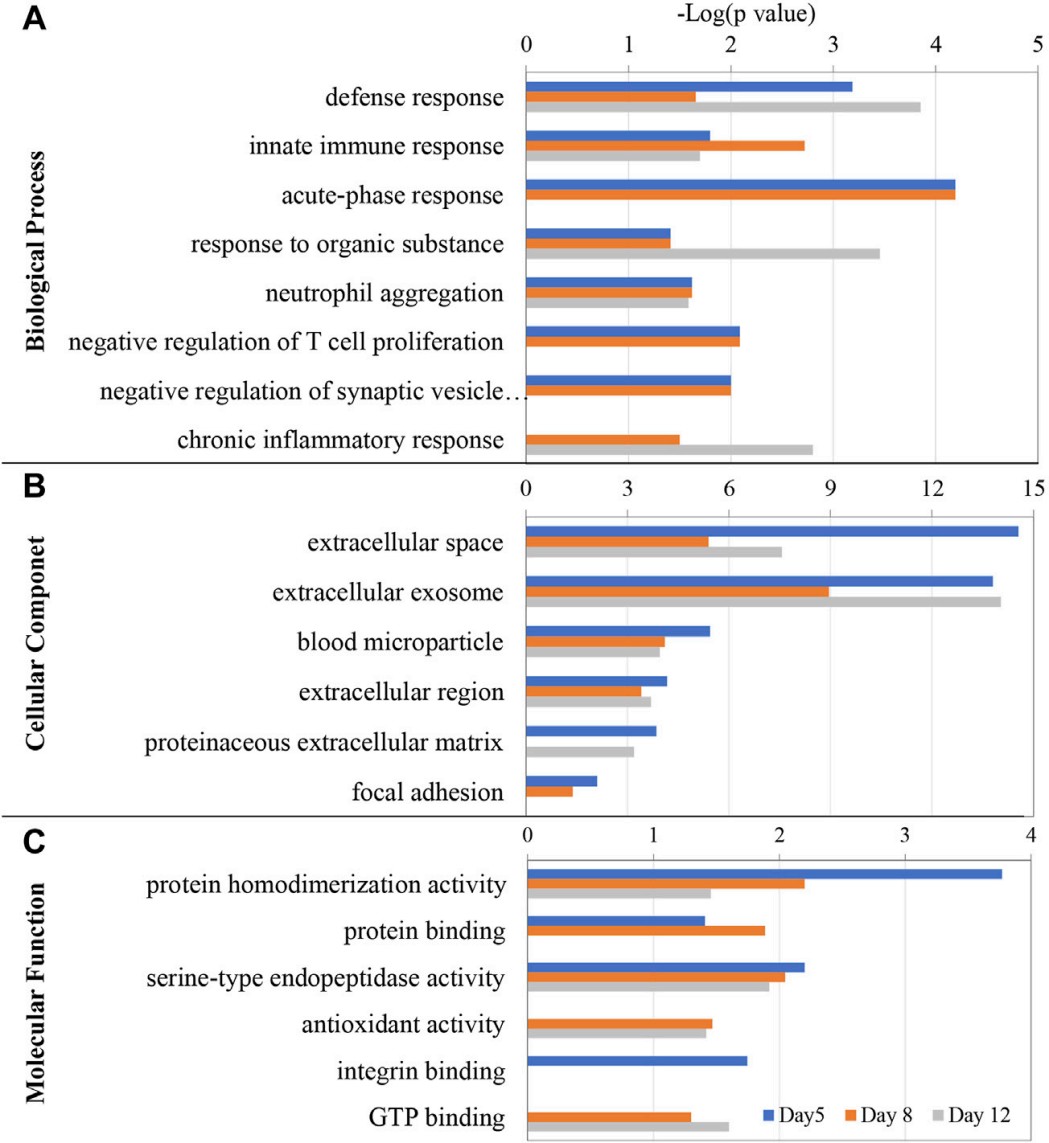
GO enrichment analysis of the differential proteins at days 5, 8, and 12 in EAU rats. **(A)** Biological process; **(B)** Cellular component; **(C)** Molecular function.

Gene ontology (GO) functional annotation was performed on the 76 differential proteins identified in EAU rats in the discovery phase. Seventy-two proteins were annotated and classified as being involved in certain biological processes (Figure 4A).

The GO enrichment analysis showed that defense response, innate immune response, neutrophil aggregation, were the main enriched biological processes at the three time points. While acute-phase response was enriched at days 5 and 8, chronic inflammatory response was only enriched at day 12. The differential proteins associated with these GO terms includes Protein S100-A8, Neutrophil antibiotic peptide NP-4, Isocitrate dehydrogenase 1 (NADP+), Annexin A2, Ras-related protein Rap-1A, Transforming protein RhoA, Heat shock protein HSP 90-alpha, Carcinoembryonic antigen-related cell adhesion molecule 1 and Glucose-6-phosphate isomerase.

In the cellular component category, most of these differential proteins were extracellular space, extracellular exosome, blood microparticle and extracellular region proteins (Figure 4B). In the molecular function category, protein homodimerization activity, serine-type endopeptidase activity, antioxidant activity, GTP binding, carbohydrate binding, protein binding and integrin binding were overrepresented on days 5 and 8. Rho GDP-dissociation inhibitor binding and protease binding were only overrepresented on day12 (Figure 4C).

### Protein-Protein Interaction Network

**FIGURE 5:**
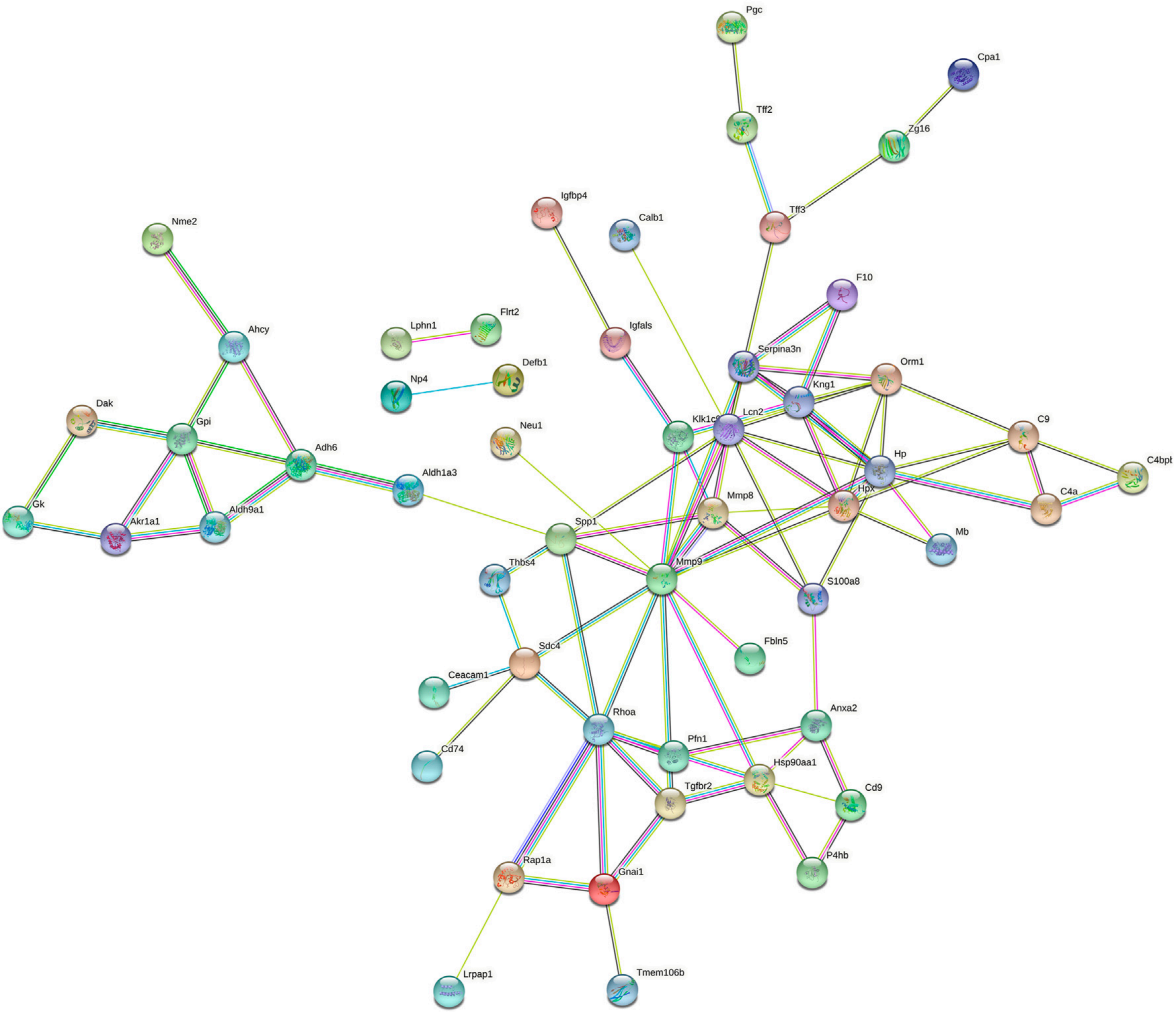
STRING PPI network analysis of the 76 differential proteins in EAU rats. The average node degree is 2.56, average local clustering coefficient is 0.404, and PPI enrichment *p*-value is < 1.0e-16.

To better understand the pathogenic mechanisms in EAU, the protein-protein interaction (PPI) network for 76 differential proteins was constructed by STRING (Figure 5). The STRING PPI network analysis exhibited that the average node degree is 2.56, the average local clustering coefficient is 0.404, and the PPI enrichment *p*-value was less than 1.0e-16. The results indicates that these differential proteins have more interactions among themselves than what would be expected for a random set of proteins of the same size and degree distribution drawn from the genome. Such an enrichment indicates that the differential proteins are at least partially biologically connected, as a group. As shown in Figure 5, many proteins were at the core of the “traffic link,” such as Adh6, Rhoa, Mmp8, S100a8, Hp, and Lcn2, which suggests that they may play an important role in the development of EAU.

### Validation of Differential Proteins in EAU Rats

Overall, 17 proteins having human orthologs (6 upregulated and 11 downregulated) were verified significantly altered at multiple time points (1.5-fold change, adjust *p* < 0.05) (Table 2). The trends of the corresponding proteins were consistent with the results from the DIA discovery phase. Twelve differential proteins changed on days 5 and 8, even when there were no clinical manifestations or histopathological ocular damage. These 12 proteins are potential biomarkers for early diagnosis of uveitis to prevent the development of visual impairment. Five differential proteins changed at three time-points and showed progressive changes as the uveitis progressed, and another five differential proteins changed only on day 12 when EAU severity peaked. These 10 proteins may serve as potential biomarkers for prognostic evaluation of uveitis.

**TABLE 2:** The details of differential urinary proteins validated by PRM analysis.

UniProt ID	Protein name	Human orthologs	Fold change			Biomarkers for diseases	Uveitis-related
			D5	D8	D12		
O88766	Neutrophil collagenase	P22894	2.20	1.95	2.40	TNBS-induced colitis (Qin et al. 2019b)	Yes
P30152	Neutrophil gelatinase-associated lipocalin	P80188	2.06	2.91	3.54	Kidney injury (Sauriasari et al. 2021), kidney toxicity (Tajima et al. 2019)	
P06866	Haptoglobin	P00738	1.55	1.58	2.01	Diabetic kidney disease (Liu et al. 2020; Zheng et al. 2021)	Yes
P27590	Uromodulin	P07911	0.61	0.49	0.54	Medication-overuse headache	
Q920A6	Retinoid-inducible serine carboxypeptidase	Q9HB40	0.61	0.46	0.38		
P02764	Alpha-1-acid glycoprotein	P02763	2.96	1.75	—	Adult-onset Still’s disease (Sun et al. 2020), Lupus nephritis (Davies et al. 2021), hepatitis B virus-associated hepatocellular carcinoma (Zhan et al. 2020)	Yes
P02767	Transthyretin	P02766	0.61	0.64	—	Ovarian Cancer (Lee et al. 2019), hyperthyroidism (Benabdelkamel et al. 2021)	
Q62930	Complement component C9	P02748	1.92	—	1.53		Yes
P01048	T-kininogen 1	P01042	1.66	—	—		
Q63530	Phosphotriesterase-related protein	Q96BW5	—	0.63	0.33		
P31211	Corticosteroid-binding globulin	P08185	—	0.66	—		
P17164	Tissue alpha-L-fucosidase	P04066	—	0.59	—		
P23785	Granulins	P28799	—	—	0.60	Prostate cancer (Fujita et al. 2017)	
P42854	Regenerating islet-derived protein 3-gamma	Q06141	—	—	0.56		
P45479	Palmitoyl-protein thioesterase 1	P50897	—	—	0.39		
Q6DGG1	Protein ABHD14B	Q96IU4	—	—	0.50	Renal transplantation (Navarrete et al. 2020)	
Q9EPF2	Cell surface glycoprotein MUC18	P43121	—	—	0.60		Yes

— Means does not reach the criteria (fold change-1.5, *p* < 0.05) compared with control group.

## Discussion

In this study, we first studied urinary protein candidate biomarkers for uveitis using DIA combined with PRM in experimental autoimmune uveitis (EAU) rats. In discovery phase, a total of 704 urinary proteins were identified via DIA proteomic technique, of which 76 were significantly changed (34, 36, and 37 on days 5, 8, and 12, respectively, after bovine IRBP immunization) (1.5-fold change, *p* < 0.05). GO analysis of the 76 differential proteins showed that the acute-phase response, innate immune response, neutrophil aggregation, and chronic inflammatory response were significantly enriched. The activity of neutrophils was increased, and there was intense neutrophil infiltration in the early stage of inflammation in uveitis (Leccese and Alpsoy, 2019; Salmaninejad et al., 2019). The acute inflammatory response, innate immune response and inflammatory response were significantly enriched at days 5 and 8. Activated innate immunity plays an important role in the pathogenesis of uveitis (Forrester et al., 2013). The PPI network of the differential proteins constructed by STRING revealed that such an enrichment indicates that the proteins are partially biologically connected in EAU as a group. These results suggest that urine proteome could sensitively reflect pathophysiological changes during the progression of EAU.

After PRM-validation, 17 proteins having human orthologs were verified significantly changed consistent with the trend in the discovery phase. Of these, eight proteins were known disease biomarkers in urine as shown in Table 2. Based on the proteome analysis, we identified 12 differential proteins on days 5 and 8 with the potential as biomarker of the early diagnosis of uveitis. Because, there are no clinical manifestations and histopathological ocular damage on days 5 and 8. Among this early diagnosis panel, four proteins were previously reported to be associated with uveitis, including Neutrophil collagenase (MMP8), Haptoglobin (HPT), Alpha-1-acid glycoprotein (AN1G), and Complement component C9 (C9), Matrix metalloproteinases (MMPs) constitute a family of zinc-dependent endopeptidases that function to maintain and remodel tissue architecture. Individual MMPs have been found to be expressed in all eye tissues (Sivak and Fini, 2002), and the expression of MMPs (MMP2, MMP8, MMP12, and MMP13) is positively associated with increased levels of corneal fibrosis and neovascularization (Wolf et al., 2019). In the aqueous humor of juvenile idiopathic arthritis patients with anterior uveitis, the levels of MMPs (MMP1, MMP3, MMP9) are significantly higher than those in controls (Bauer et al., 2018). In retinas from EAU rats, the expression of MMPs (MMP7, MMP8, MMP12) was increased compared with that in naive controls (Wallace et al., 1999). In addition, MMP8 has been found to be differentially changed in the urine of individuals with autoimmune inflammatory diseases, such as experimental autoimmune myocarditis (Zhao et al., 2018). In a trinitrobenzene sulfonic acid (TNBS)-induced colitis rat model, the level of MMP8 is reported to be elevated about 3-fold in urine (Qin et al., 2019a). HPT and C9 are reported to be closely associated with Behcet’s disease (BD). Serum C9 is significantly increased in BD patients with relapse (Lehner and Adinolfi, 1980); and higher serum HPT levels are detected in BD patients compared to controls, and there is no obvious difference between the quiescent and active stages (Mao et al., 2008; Yalcindag et al., 2008). And increased serum HPT is also observed in EAU rats compared to controls (Guo et al., 2015). Alpha-1-acid glycoprotein plays a key role in modulating the activity of the immune system during the acute-phase reaction. Interestingly, we also found it significantly increased in the tears of the active BDU eye compared to the contralateral quiescent eye (Liang et al., 2020).

Five differential proteins changed at three time-points and showed progressive changes as the uveitis progressed, and another five differential proteins changed only on day 12 when EAU severity peaked. These 10 proteins may serve as potential biomarkers for prognostic evaluation of uveitis. Among these, cell surface glycoprotein MUC18 (CD146) was previously reported to be associated with uveitis. CD146 is a transmembrane glycoprotein expressed at the junction of endothelial cells (Bardin et al., 2001). It plays an important role in angiogenesis and vessel remodeling (Bardin et al., 2001; Yan et al., 2003). In age-related macular degeneration patients, the serum CD146 level is significantly higher than that in controls (Liu et al., 2019).

The presented work was a preliminary study, indicating the feasibility of urine as a potential diagnostic biomarker approach for uveitis. Uveitis is a common, sight-threatening inflammatory ocular disease and includes multiple heterogeneous clinical entities, such as Behcet disease (BD), sarcoidosis, Vogt–Koyanagi–Harada (VKH) and ankylosing spondylarthritis. However, only a small number of IRBP-induced EAU rats were included. We are well aware of the limited of EAU rat model and the lack of clinical validation, which is necessary for future study. To further evaluated the sensitivity and specificity of these differential proteins, urine samples from uveitis patients should be included in further studies. In addition, an in-depth profile of the urine proteome may provide a more comprehensive and accurate understanding of uveitis.

## Conclusion

In summary, our results revealed that the urinary proteome could reflect dynamic pathophysiological changes in EAU. These differential proteins could be potential biomarkers for the early diagnosis or prognostic evaluation of uveitis. Which also provides valuable clues for investigating the pathogenic mechanisms of uveitis.

## Data Availability

Raw data were uploaded to iProX. URL: https://www.iprox.cn/page/PSV023.html;?url=1643435018300wvph, password: SA4M.
